# Heat Stress-Induced Multiple Multipolar Divisions of Human Cancer Cells

**DOI:** 10.3390/cells8080888

**Published:** 2019-08-13

**Authors:** Shaoyong Chen, Mingyue Liu, Huiming Huang, Bo Li, Hucheng Zhao, Xi-Qiao Feng, Hong-Ping Zhao

**Affiliations:** Institute of Biomechanics and Medical Engineering, AML, Department of Engineering Mechanics, Tsinghua University, Beijing 100084, China

**Keywords:** heat stress, multipolar divisions, centrosome aberration, polyploidy, mitotic slippage, cytokinesis failure, cell fusion, tumor heterogeneity

## Abstract

Multipolar divisions of heated cells has long been thought to stem from centrosome aberrations of cells directly caused by heat stress. In this paper, through long-term live-cell imaging, we provide direct cellular evidences to demonstrate that heat stress can promote multiple multipolar divisions of MGC-803 and MCF-7 cells. Our results show that, besides facilitating centrosome aberration, polyploidy induced by heat stress is another mechanism that causes multipolar cell divisions, in which polyploid cancer cells engendered by mitotic slippage, cytokinesis failure, and cell fusion. Furthermore, we also find that the fates of theses polyploid cells depend on their origins, in the sense that the polyploid cells generated by mitotic slippage experience bipolar divisions with a higher rate than multipolar divisions, while those polyploid cells induced by both cytokinesis failure and cell fusion have a higher frequency of multipolar divisions compared with bipolar divisions. This work indicates that heat stress-induced multiple multipolar divisions of cancer cells usually produce aneuploid daughter cells, and might lead to genetically unstable cancer cells and facilitate tumor heterogeneity.

## 1. Introduction

Normal bipolar mitosis in eukaryotic cells is an essential process in the development of tissues and organs. It maintains genomic stability by establishing bipolar spindles with an accurate centrosome number and centrosome structure during cell division, and executes precise segregation of chromosomes, centrosomes, cytoplasm, and organelles during the mitosis for mammalian cells. Failure to properly duplicate the centrosome number, such as centrosome overduplication or amplification, can lead to spindle defects, aneuploidy, cell polarity disruption, and unequal segregation of chromosomes, and is recognized as a key remark of many human cancers [1,2]. Meanwhile, multiple centrosomes in cancer cells might promote cell malignancy by enhancing the potential of multipolar mitoses [3,4,5,6].

Multipolar cell division can be promoted by polyploid cells [7], and various treatments by paclitaxel [8], colchicine [9], carboplatin [10], confined microenvironment [11,12], UV- and X-radiation [13], and even heat stress [14,15,16,17,18,19,20]. Through time lapse observation, Temme et al. [14] found that the fraction of dividing cells that exhibited multipolar divisions was elevated to about 50% in the heated Chinese hamster ovary (CHO) cells. Vidair et al. [15] showed 76% of heated CHO cells contained multipolar spindles in prophase-metaphase, while such abnormalities were observed less than 3% in control cells; they further revealed that multipolar mitotic spindles in CHO cells were related with multiple foci of the microtubule nucleating material induced by heat treatment. In addition, Gupta et al. [16] showed that about 5% of near-tetraploid embryonal carcinoma cells underwent multipolar mitosis, while about 67% of the mitotic cells showed multipolar mitosis with 48 h recovery after heat shock, reduced significantly at 96 h recovery time with about 40% of mitotic cells undergoing multipolar mitosis. Their results demonstrated that multipolar divisions induced by heat shock triggered with centrosome amplification, and further chromosome missegregation in near-tetraploid embryonal carcinoma cells, thereby having a risk in triggering aneuploidy and chromosomal instability. Nakahata et al. [17] treated three normal human diploid cell lines (HE27, HE49, and HE51) and five human cancer cell lines (T24, NC1-H1299, HT1080, HeLa, and MCF-7) with 43 ℃ for 2 h, respectively. They found that abnormal centrosomes in tumor cells were induced and significant numbers of mitotic cells with multiple poles appeared, while not in normal human diploid cells. Furthermore, Petrova et al. [18] found that HeLa cells can escape from heat stress-induced cellular G2 arrest, and the mitosis they enter is multipolar due to amplified centrosomes. Meanwhile, Zhang et al. [19] demonstrated that heat stress can also drive fish embryonic cells to form multipolar spindles in their mitosis, which was assumed to be caused by insufficient disorganization of daughter centrioles and splitting from the mother centriole by heat shock. Baek et al. [20] concluded that heat shock can induce de novo centriole assembly in mammalian cells, thus causing centrosome amplification. These previous studies showed that subcellular mechanisms underlying heat stress-induced multipolar divisions can be attributed to the structural or numerical aberrations in centrosomes directly stemming from heat stress.

About a century ago, some plant cells were observed to contain more than two copies of chromosomes [21]. Many eukaryote cells have also been recognized as polyploid cells in diverse circumstances [22]. It has been widely accepted that polyploid cells can be formed with different biological routes, such as mitotic slippage, cytokinesis failure, and cell fusion with doubling centrosomes, and even with centrosome aberrations, compared with normal diploid cells [23,24,25]. Rabbit egg cells exposed briefly to supra-normal temperature were found to form tetraploid mammalian cells by sufficiently inhibiting cell division without markedly affecting chromosome division [26]. Polyploidy, or tetraploidy may also emerge in mouse egg cells after the application of heat shock at the time of first cleavage metaphase to suppress normal division [27]. Meanwhile, human epidermal cells can also generate tetraploidy after heat shock [28]. Subsequent studies revealed that human cells in mitosis exposed to heat shock can result in polyploid or tetraploid cells by directly entering interphase without completing division due to the disassembled spindle and destroyed contractile ring [29,30,31]. Tan et al. [32] found that that heat shock causes polyploidization of human colon carcinoma cell lines by mitotic exit without chromosome segregation. Furthermore, cytokinesis failure can generate tetraploidy that can undergo multipolar divisions and promote tumorigenesis in p53-null cancer cells [33,34]. In addition, cell fusion is another mechanism for cancer cells to exhibit a higher degree of aneuploidy [35,36]. The above polyploid cancer cells and their aneuploid daughter cells caused by heat treatment may induce tumor heterogeneity and further drug resistance [32,37,38]. Since heat stress can lead to alterations in centrosomes and different abnormal biological routes, such as mitotic slippage, cytokinesis failure, or cell fusion, it can be directly observed and responsible for the formation of polyploid cells after heat stress treatment.

To identify the thermal target which induces multipolar divisions from the triggered polyploid cells in human cancer cells, direct observation of abnormal biological routes induced by heat stress is required using a long-term real-time live cell imaging system. As is well known, breast cancer is the leading cancer in women, and gastric cancer is another leading cause of cancer-related mortality all around the world. In addition, hyperthermia has been used as a routine method in clinical treatments of breast cancer and gastric cancer [39,40]. In this study, therefore, we select human gastric carcinoma MGC-803 cells and human breast cancer MCF-7 cells to track the heat stress-induced mitosis process using long-term live cell imaging. The defects in centrosomes of the heated cancer cells are detected with immunofluorescence staining. The multipolar division frequencies along different biological routes induced by heat stress are then obtained. Meanwhile, cell proliferation inhibition by heat shock is tested by MTT assay, and cell cycle analysis is performed by flow cytometry. Our results show that MGC-803 cells and MCF-7 cells with heat stress treatment can exhibit mitotic slippage, cytokinesis failure, cell fusion, and centrosomes aberrations simultaneously, producing multiple multipolar divisions. Furthermore, compared with heat-induced centrosome aberrations, we propose that most of the heated human cancer cells undergoing multipolar divisions would primarily suffer polyploidization by mitotic slippage, cytokinesis failure and cell fusion. In addition, the fates of theses heated polyploid cells are also closely associated with their origins.

## 2. Materials and Methods

### 2.1. Cell Culture

MGC-803 cells and MCF-7 cells were obtained from National Infrastructure of Cell Line Resource of China (5 Dong Dan San Tiao, Beijing, China). MGC-803 cell line was further authenticated with short tandem repeat (STR) profiling in the National Infrastructure. MGC-803 and MCF-7 cells were maintained in Roswell Park Memorial Institute 1640 (RPMI 1640, Gibco, New York, USA) medium and high glucose Dulbecco’s modified Eagle’s medium (DMEM, Gibco, New York, USA), respectively, supplemented with 10% fetal bovine serum (Gibco, New York, NY, USA), 100 U/mL penicillin (Solarbio, Beijing, China), and 100 mg/mL streptomycin (Solarbio, Beijing, China). Cells were cultured at 37 °C in a humidified atmosphere of 95% air and 5% CO_2_.

### 2.2. Heat Stress Treatment

Exponentially growing cells seeded in Falcon culture dishes (35 × 10 mm style) were heat stress-treated at 48 °C for 10 min [41,42] in a temperature-controlled water bath. Fresh culture medium was exchanged into each dish after heat shock treatment, and the cells were returned to normal culture conditions for further observation.

### 2.3. MTT Assay

Effects of heat stress treatment on cancer cell viability were determined using the 3-(4,5-Dimethyl-2-thiazolyl)-2,5-diphenyl-2H-tetrazolium bromide (MTT) assay. Cells were seeded in 6 replicates in a 96-well plate at a concentration of 5 × 10^3^ cells/well with 200 μL fresh culture medium, and allowed to adhere overnight. MTT reagent (Thiazolyl Blue Tetrazolium Bromide, Sigma, Missouri, USA) was dissolved at a concentration of 5 mg/mL in PBS and filtered with a 0.45-μm filter. The MTT solution (20 μL) was added to each well, including the control well without cells. After incubation for 4 h, the medium was then removed, and 180 μL of DMSO was subsequently added to each well to dissolve the formazan crystals resulting from MTT reduction. Absorbance intensities were measured at 490 nm with a plate reader (Model 550, BioRad, Hercules, CA, USA).

### 2.4. Flow Cytometry

Cell cycle analysis was carried out by flow cytometry (BD, FACSAria II, San Jose, CA, USA) as described in a previous paper [43]. Cells were seeded into culture dishes with a density of 2 × 10^4^ cells/well for 24 h. At different recovery time points after heat stress treatment, attached cells were trypsinized, harvested, and washed with phosphate-buffered saline (PBS). Thereafter, the cells were fixed with ice-cold 70% ethanol for 24 h and stained with a propidium iodide solution (50 μg/mL) supplemented with RNase A (100 μg/mL) (Beyotime Biotechnology, Shanghai, China) at 37 °C for 30 min. The cells were analyzed using flow cytometry and FlowJo software.

### 2.5. Long-Term Live Imaging

To observe the cell shapes and aberrant cell divisions induced by heat stress, the cells were subjected to live imaging by an Olympus IX83 microscope equipped with a cooled CCD camera, a stage-top microscope incubator maintained at 37 °C with 5% CO^2^ (Tokai Hit, Shizuoka, Japan). Cell images were taken every 5 min immediately after heat shock. The cell number in each image was determined and their edges were traced manually by using the NIH Image J software. The variations in the cell number, spreading area, and aspect ratio (the length/width of a cell) [44] were measured. In addition, multipolar divisions per cell cycle were analyzed through long-term live cell imaging. The frequency of multipolar division was calculated by the number of multipolar cell divisions divided by that of the total cell divisions.

### 2.6. Immunofluorescence Assay

The cells grown on coverslips were heat-treated and incubated at 37 °C for 72 h. Subsequently, the cells were fixed with 4% formaldehyde for 10 min, washed with PBS and permeabilized with 0.1% Triton X-100 in PBS for 10 min at room temperature. Furthermore, the samples were blocked with 5% BSA in PBS for 60 min. After washing 3 times in PBS, the cells were incubated with primary antibodies overnight at 4 °C. Primary antibodies included rabbit anti-alpha tubulin (ab52866, Abcam, Cambridge, MA, USA) and mouse anti-pericentrin (ab28144, Abcam, Cambridge, MA, USA). Then, the cells were washed 3 times in PBS, followed by sequential incubation with secondary antibodies for 60 min at room temperature. Secondary antibodies included Goat anti-Rabbit IgG (H&L) conjugated with Alexa Fluor^®^ 594 (ab150080, Abcam, Cambridge, MA, USA) and Goat anti-Mouse IgG (H&L) conjugated with Alexa Fluor^®^ 488 (ab150113, Abcam, Cambridge, MA, USA). Finally, coverslips were mounted in PBS containing 1 μg/mL DAPI for 10 min for DNA staining. The stained cells were visualized and analyzed using a laser scanning confocal microscope (LSM780, Zeiss, Germany) with a 63X/1.4NA oil immersion objective lens and excitation wavelengths of 405 nm, 488 nm and 594 nm. All images were acquired using ZEN 2012 software.

### 2.7. Statistical Analysis

Data were expressed as mean ± SD subjected to the t-test. Data differences were considered to be statistically significant when the *p*-value was less than 0.05. All statistical analyses were performed using SPSS version 19.0.

## 3. Results

### 3.1. Biological Responses of Human Cancer Cells to Heat Stress

**Figure 1:**
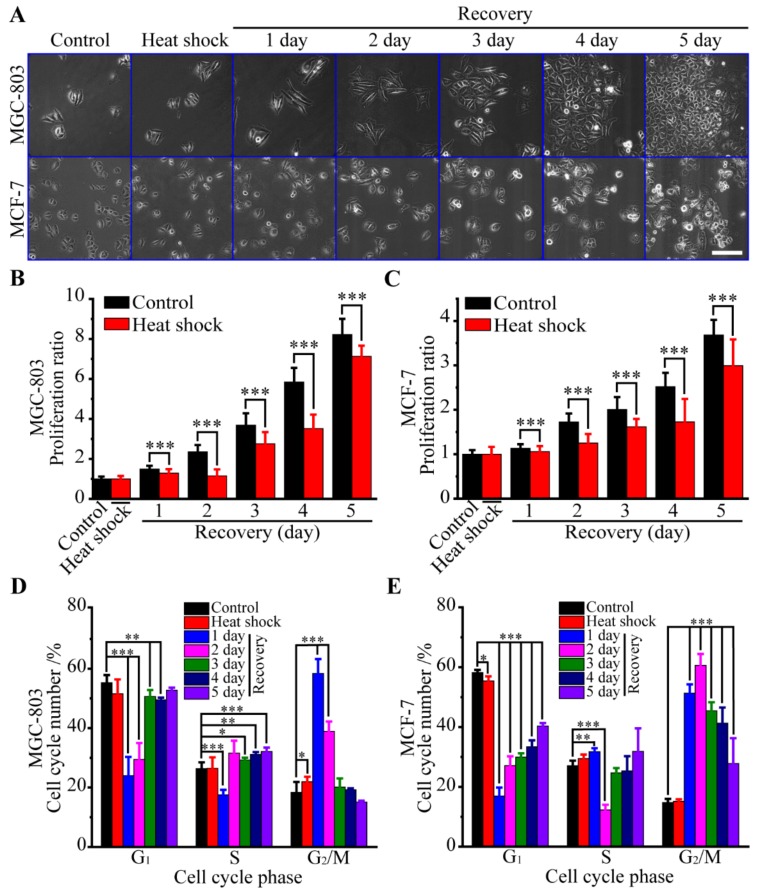
Biological responses of human cancer cells caused by heat stress. (**A**) Time-lapse images of MGC-803 and MCF-7 cells (scale bar: 200 μm). (**B**,**C**) Cell proliferations of MGC-803 and MCF-7 cells, respectively (mean values ± SD are shown from three independent experiments). (**D**,**E**) Cell cycles of MGC-803 and MCF-7 cells, respectively (mean values ± SD are shown from three independent experiments). *p*-value < 0.05 was considered statistically significant, *p < 0.05, **p < 0.01, and ***p < 0.001.

We first examine the biological responses of human cancer cells to heat stress. Firstly, the morphologies of MGC-803 and MCF-7 cells at different recovery times are shown in Figure 1A with phase-contrast micrographs, and further statistical analysis of cell morphologies is shown in Figure S1. When exposed to heat shock, MGC-803 cells immediately become more slender and then gradually recover to their original morphology, while the impact of heat stress on morphologies of MCF-7 cells is unremarkable. For example, the average aspect ratio of MGC-803 cells is 1.8 times larger than that of the control group at 24 h after heat shock. Meanwhile, the average spreading area of the MGC-803 cells first decreases immediately when exposed to heat shock, and gradually increases up to 5459 μm^2^ at 48 h after heat shock, and then decreases to the normal size due to cell division. The average spreading area of MCF-7 cells increases as a function of recovery time and levels off at a saturating level of 2300–2458 μm^2^, and then decreases at 120 h after heat shock.

Secondly, the effects of heat shock treatment on the viability of cancer cells are determined using MTT assay. The proliferation ratio is defined as the percentage of viable cells at different recovery time points relative to the control cells. From Figure 1B,C the MTT assay of cancer cells during heat stress and recovery reveals that heat shock compromised the proliferations of MGC-803 and MCF-7 cell lines during the recovery phase, compared with the control groups. Furthermore, the proliferation ratios of the MGC-803 cell line with and without heat stress are larger than those of the MCF-7 cell line, indicating MGC-803 cells are growing more vigorously than MCF-7. This observation is also consistent with the results in Figure 1A.

Thirdly, flow cytometry is employed to examine the cell cycle distributions of MGC-803 and MCF-7 cells with and without heat stress treatment. The obtained flow cytometry histograms of MGC-803 and MCF-7 cells with different recovery time after heat stress are shown in Figure S2. Our results from flow cytometry analysis show that heat stress exposure has a significant impact on cell cycle distribution at recovery time, and the change in G_1_ and G_2_/M phases is quite obvious, as shown in Figure 1D,E, that is, heat stress significantly reduced the cell number in G_1_ and S phases but remarkably increased the cell number in G_2_/M phase. Then, the cell numbers in G_1_, S, and G_2_/M phases had trends to gradually return to normal with recovery time, and these trends were dependent on cell lines. In other words, heat exposure arrested the cell cycles of MGC-803 and MCF-7 cells in G_2_/M phases. With the increased recovery time after heat stress, cancer cells gradually escaped from the cell cycle arrest and cell divisions were observed.

### 3.2. Multipolar Divisions Induced by Heat Stress

Implementation of a long-term live cell imaging approach shed a new light on the process of mitotic division of human cancer cells after heat shock treatment. Multipolar division origins and their outcomes induced by heat stress were then investigated by reverse examination of these time-lapse records.

#### 3.2.1. The Route of Mitotic Slippage

**Figure 2:**
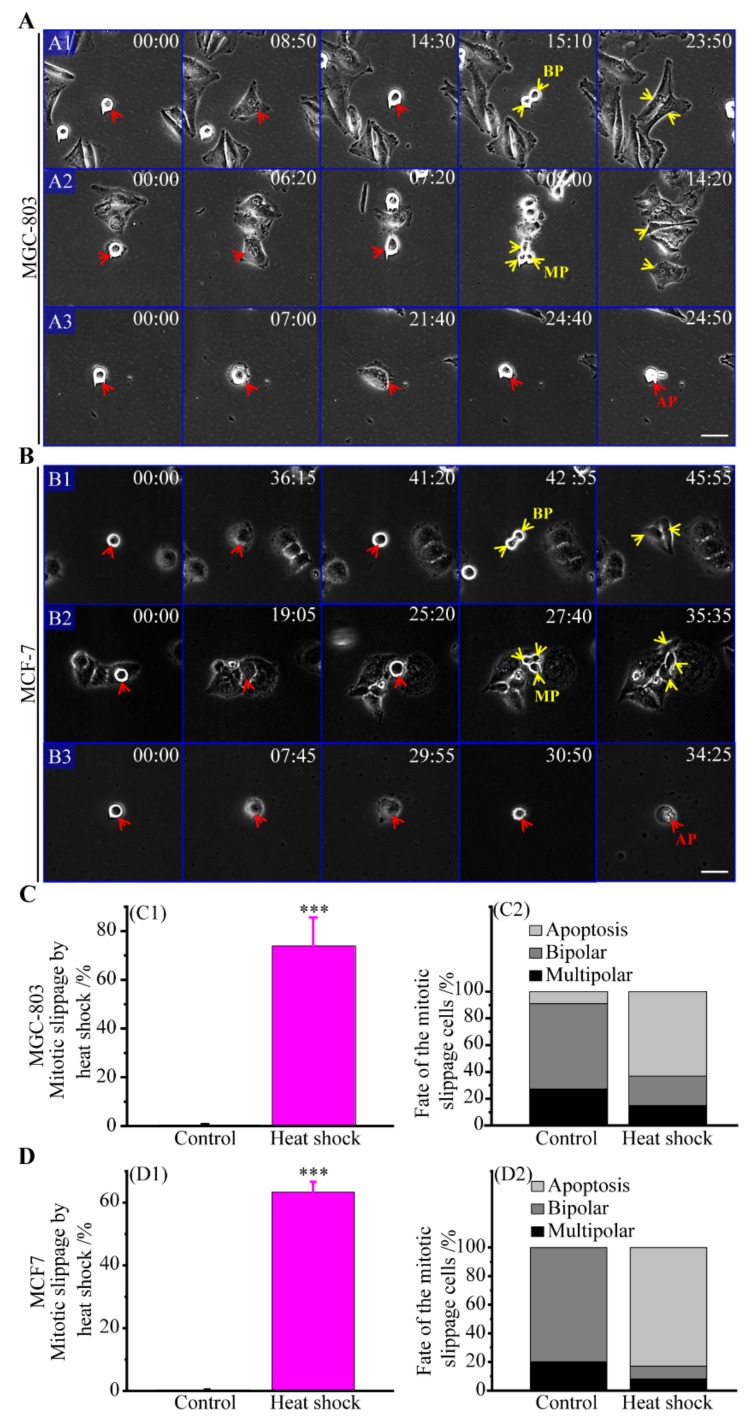
Mitotic slippage of cancer cells caused by heat stress and their cell fates. (**A**) Time-lapse images of MGC-803 cells; (**A1**) bipolar division, (**A2**) multipolar division, and (**A3**) cell apoptosis of MGC-803 cells after mitotic slippage induced by heat stress, scale bar: 50 μm. (**B**) Time-lapse images of MCF-7 cells; (**B1**) bipolar division, (**B2**) multipolar division, and (**B3**) cell apoptosis of MCF-7 cells after mitotic slippage induced by heat stress, scale bar: 40 μm. (**C**) The mitotic slippage frequency of MGC-803 cells and their cell fates; (**C1**) the frequency of MGC-803 cells with mitotic slippage (mean values ± SD are shown from three independent experiments); (**C2**) MGC-803 cells fates with mitotic slippage; the ratios through the routes of bipolar divisions, multipolar divisions and cell apoptosis are 7:3:1 in the control and 67:45:191 in the heat shock group of MGC-803 cells, respectively. (**D**) The mitotic slippage frequency of MCF-7 cells and their cell fates; (**D1**) the frequency of MCF-7 cells with mitotic slippage (mean values ± SD are shown from three independent experiments); (**D2**) MCF-7 cells fates with mitotic slippage; the ratios through the routes of bipolar divisions, multipolar divisions and cell apoptosis are 4:1:0 in the control and 11:10:102 in the heat shock group of MCF-7 cells, respectively. BP (bipolar), MP (multipolar), and AP (apoptosis). *p*-value < 0.05 was considered statistically significant, ***p < 0.001.

When exposed to heat shock, the cancer cells were in either interphase or mitosis state (Figure S3). The cancer cells in interphase were rarely observed to carry out mitotic slippage (Figure S4). In this study, therefore, only the cells in mitosis state were applied to count the mitotic slippage frequencies induced by heat shock. Approximately 73.9% of MGC-803 and 63.3% of MCF-7 cells in mitosis were observed directly undergoing mitotic slippage after the mitotic cells had experienced the heat stress (Figure 2A,B,(C1,D1)), while almost MGC-803 (99.3%) and MCF-7 (98.2%) mitotic cells in controls divided normally. In fact, mitotic slippage is spontaneous but a rare event in human cancer cells, and it can be efficiently elevated by heat stress. The heated cells in mitosis directly enter the next cell cycle G1 phase without completing divisions, thereby leading to polyploid cells with multifold chromosomes and centrosomes [24,25,45]. These polyploid cells then underwent bipolar divisions, multipolar divisions and cell apoptosis, as also shown in Figure 2A,B. As shown in Figure 2(C2,D2), our results revealed that the shares of bipolar divisions, multipolar divisions and cell apoptosis of these polyploid cells induced by heat stress were about 21.1%, 14.9%, and 63.0% in MGC-803 cells, and 9.0%, 8.1%, and 82.9% in MCF-7 cells, respectively. In the control group, the shares of bipolar divisions, multipolar divisions and cell apoptosis of these polyploid cells were about 63.6%, 27.3%, and 9.1% in MGC-803 cells, and 80.0%, 20.0% and 0% (not found) in MCF-7 cells, respectively. Hence, we can make the following conclusions. Firstly, the apoptosis rate of these polyploid cells generated by mitotic slippage caused by heat stress was much higher than that generated by spontaneous mitotic slippage in the control group. Secondly, these polyploid cells generated by mitotic slippage experience multipolar divisions at a lower frequency than bipolar divisions in both the control and the heat stress groups. Thirdly, the progeny generated by bipolar divisions of heated cancer cells after mitotic slippage also have the potential to undergo multipolar divisions (Figures S5A and S5B). For example, we tracked 36 progenies from those bipolar divisions after mitotic slippage in MGC-803 cells and we found 14, 9, and 13 of these daughter cells undergoing bipolar divisions, multipolar divisions and cell apoptosis, respectively (Figure S5C).

#### 3.2.2. The Route of Cytokinesis Failure

**Figure 3:**
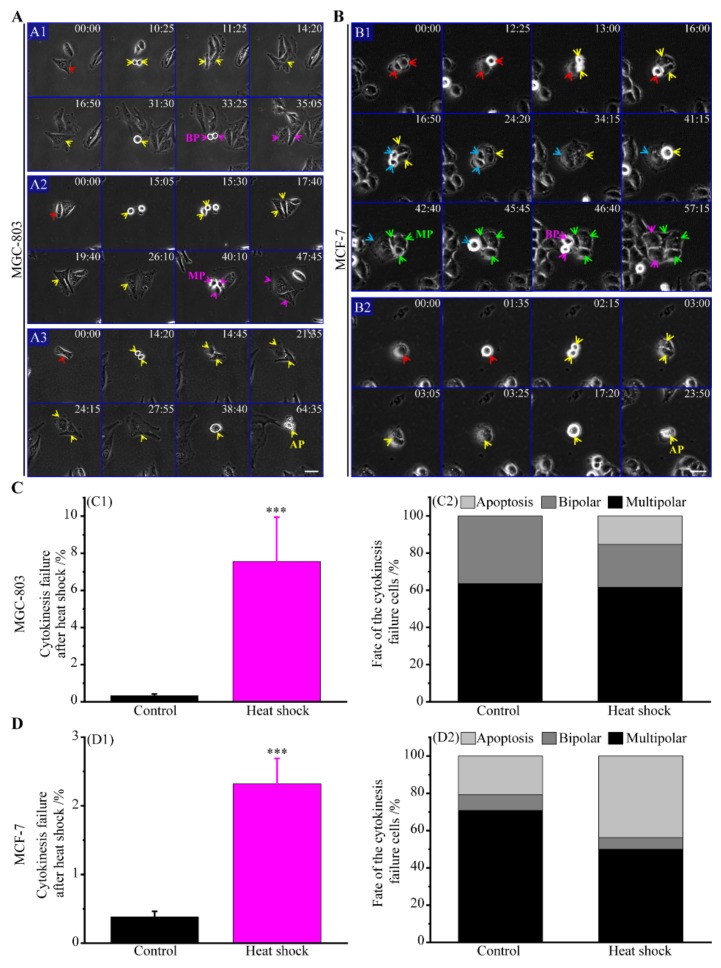
Cytokinesis failure of cancer cells caused by heat stress and their cell fates. (**A**) Time-lapse images of MGC-803 cells; (**A1**) bipolar division, (**A2**) multipolar division, and (**A3**) cell apoptosis of MGC-803 cells after cytokinesis failure induced by heat stress, scale bar: 50 μm. (**B**) Images of MCF-7 cells; (**B1**) bipolar division, (**B2**) multipolar division, and (**B3**) cell apoptosis of MCF-7 cells after cytokinesis failure induced by heat stress, scale bar: 40 μm. (**C**) The cytokinesis failure frequency of MGC-803 cells and their cell fates; (**C1**) the frequency of MGC-803 cells with cytokinesis failure (mean values ± SD are shown from three independent experiments); (**C2**) MGC-803 cells fates with cytokinesis failure; the ratios through the routes of bipolar divisions, multipolar divisions, and cell apoptosis are 4:7:0 in the control and 9:24:6 in the heat shock group of MGC-803 cells, respectively. (**D**) The cytokinesis failure frequency of MCF-7 cells and their cell fates; (**D1**) the frequency of MCF-7 cells with cytokinesis failure (mean values ± SD are shown from three independent experiments); (**D2**) MCF-7 cells fates with cytokinesis failure; the ratios through the routes of bipolar divisions, multipolar divisions and cell apoptosis are 2:17:5 in the control and 1:8:7 in the heat shock group of MCF-7 cells, respectively. BP (bipolar), MP (multipolar), and AP (apoptosis). *p*-value < 0.05 was considered statistically significant, *** *p* < 0.001.

When heat stress was applied to cancer cells in interphase state, some of the cells undergone bipolar divisions failed at cytokinesis (Figure 3A,B). The spontaneous cytokinesis failure in MGC-803 and MCF-7 cells in the control groups was about 0.3% and 0.4%, while the rate was elevated to 7.6% and 2.3% by heat stress, respectively (Figure 3(C1,D1)). Furthermore, as shown in Figure 3(C2), the shares of bipolar divisions, multipolar divisions, and cell apoptosis of the polyploid MGC-803 cells produced by cytokinesis failure [23,24,25,45] were 36.4%, 63.6%, and 0% in the control group and 23.1%, 61.5%, and 15.4% in the heat shock group, respectively. For MCF-7 cells, Figure 3(D2) shows that the shares of bipolar divisions, multipolar divisions and cell apoptosis of the polyploid cells produced by cytokinesis failure were 8.4%, 70.8%, and 20.8% in the control group and 6.3%, 50.0%, and 43.7% in the heat shock group, respectively. It is worth mentioning that, when heat stress was applied to the cancer cells just in the cytokinesis state, polyploidy would also be produced and theses polyploid cells would also be resulted in multipolar divisions (Figure S6), while this polyploidy formation is rare due that cancer cells were exposed to heat stress only 10 min in our experiments. Thus, the following conclusions can be acquired. First, the apoptosis rate of these polyploid cells generated by cytokinesis failure in the heat-stress group was higher than that in the control group. Second, these polyploid cells produced by cytokinesis failure exhibited a much higher frequency of multipolar divisions than that of bipolar divisions. Third, these polyploid cells induced by cytokinesis failure had a higher frequency of multipolar divisions than those generated by mitotic slippage.

#### 3.2.3. The Route of Cell Fusion

**Figure 4:**
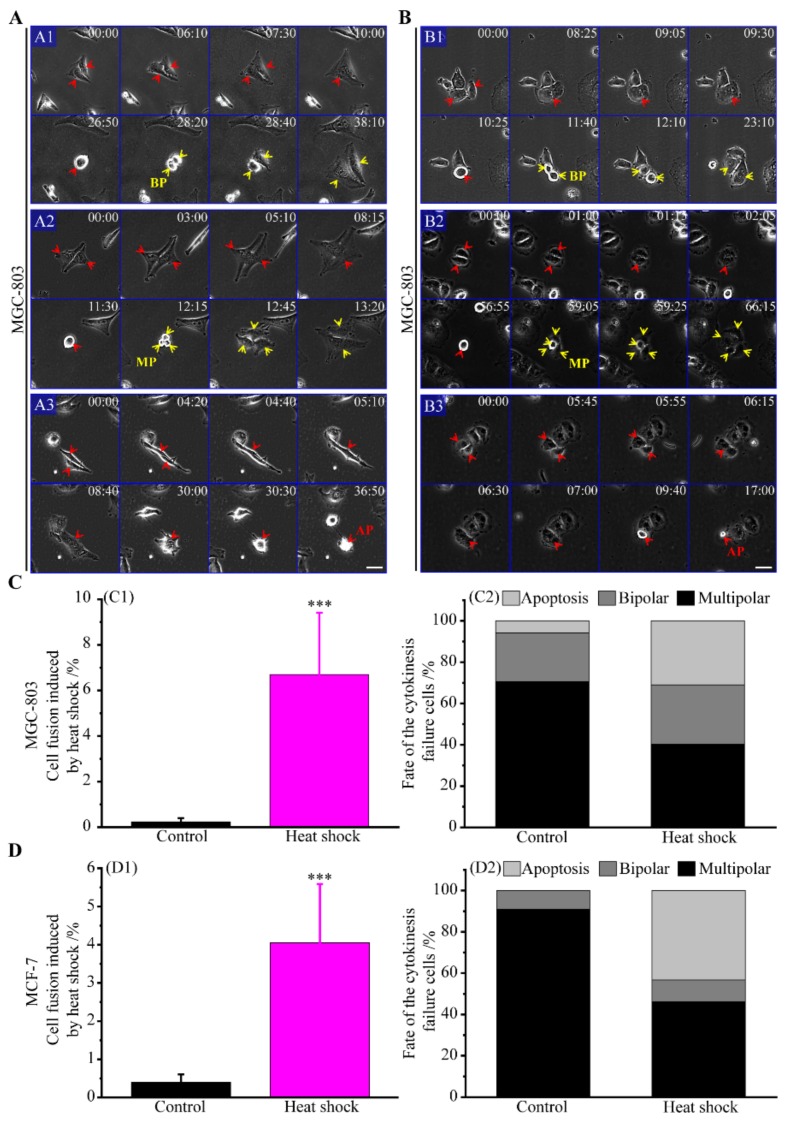
Cell fusion of cancer cells caused by heat stress and their fates. (**A**) Time-lapse images of MGC-803 cells; (**A1**) bipolar division, (**A2**) multipolar division, and (**A3**) cell apoptosis of MGC-803 cells after cell fusion induced by heat stress, scale bar: 50 μm. (**B**) Time-lapse images of MCF-7; (**B1**) bipolar division, (**B2**) multipolar division, and (**B3**) cell apoptosis of MCF-7 cells after cell fusion induced by heat stress, scale bar: 40 μm. (**C**) The cell fusion frequency of MGC-803 cells and their cell fates; (**C1**) the frequency of MGC-803 cells with cell fusion (mean values ± SD are shown from three independent experiments); (**C2**) MGC-803 cells fates with cell fusion; the ratios through the routes of bipolar divisions, multipolar divisions and cell apoptosis are 4:12:1 in the control group and 25:34:27 in the heat shock group of MGC-803 cells, respectively. (**D**) The cell fusion frequency of MCF-7 cells and their cell fates; (**D1**) the frequency of MCF-7 cells with cell fusion (mean values ± SD are shown from three independent experiments); (**D2**) MCF-7 cells fates with cell fusion; the ratios through the routes of bipolar divisions, multipolar divisions and cell apoptosis are 1:10:0 in the control and 7:31:29 in the heat shock group of MCF-7 cells, respectively. BP (bipolar), MP (multipolar), and AP (apoptosis). *p*-value < 0.05 was considered statistically significant, ***p < 0.001.

Since cell–cell contact is necessary for cell fusion, we pay attention to the pairs of cancer cells (Figure S7A,B). Normally, most cell pairs divided and proliferated without cell fusion (Figure S8). When the cell pairs were exposed to heat stress, cell proliferation was inhibited and some cells directly died, while about 78.4% of MGC-803 and 39.0% of MCF-7 cell pairs remained alive (Figure S7C). Subsequently, some alive cell pairs fused gradually (Figure 4A,B). The average duration time of cell fusion was determined to be about 123.5 min for MGC-803 and 46.9 min for MCF-7 cell pairs (Figure S9).

Cell fusion of MGC-803 and MCF-7 cell pairs rarely occurred spontaneously, at a rate of about 0.2% and 0.4%, whereas it reached a frequency of 6.7% and 4.1% after heat shock, respectively (Figure 4(C1,D1)). Similarly, cell fusion directly yielded polyploidy in human cancer cells [23,24,25,45]. We tested the fates of polyploid cells produced by cell fusion. As shown in Figure 4(C2,D2), our results revealed that, of these polyploid cells of MGC-803, 23.5% underwent bipolar division, 70.6% multipolar division and 5.5% cell apoptosis in the control group, while 28.7% underwent bipolar division, 40.3% multipolar division, and 31% cell apoptosis in the heated group. Of the polyploid cells of MCF-7, 9.1% underwent bipolar division and 90.9% underwent multipolar division in the control group, while 10.4% underwent bipolar division, 46.3% multipolar division, and 43.3% cell apoptosis in the heated group. The results on the fates of polyploidy produced by cell fusion were concordant with those by cytokinesis failure. First, the apoptosis rate of these polyploid cells generated by cell fusion caused by heat stress was higher than that generated by spontaneous cell fusion in the control group. Second, these polyploid cells generated by cell fusion exhibited a much higher frequency of multipolar divisions than that of bipolar divisions. Third, these polyploid cells generated by cell fusion had a higher frequency of multipolar divisions compared with these generated by mitotic slippage. Additionally, we can infer that the polyploid cells generated by cytokinesis failure and cell fusion have more individual nuclei that those from mitotic slippage.

#### 3.2.4. The Route of Centrosome Aberration

**Figure 5:**
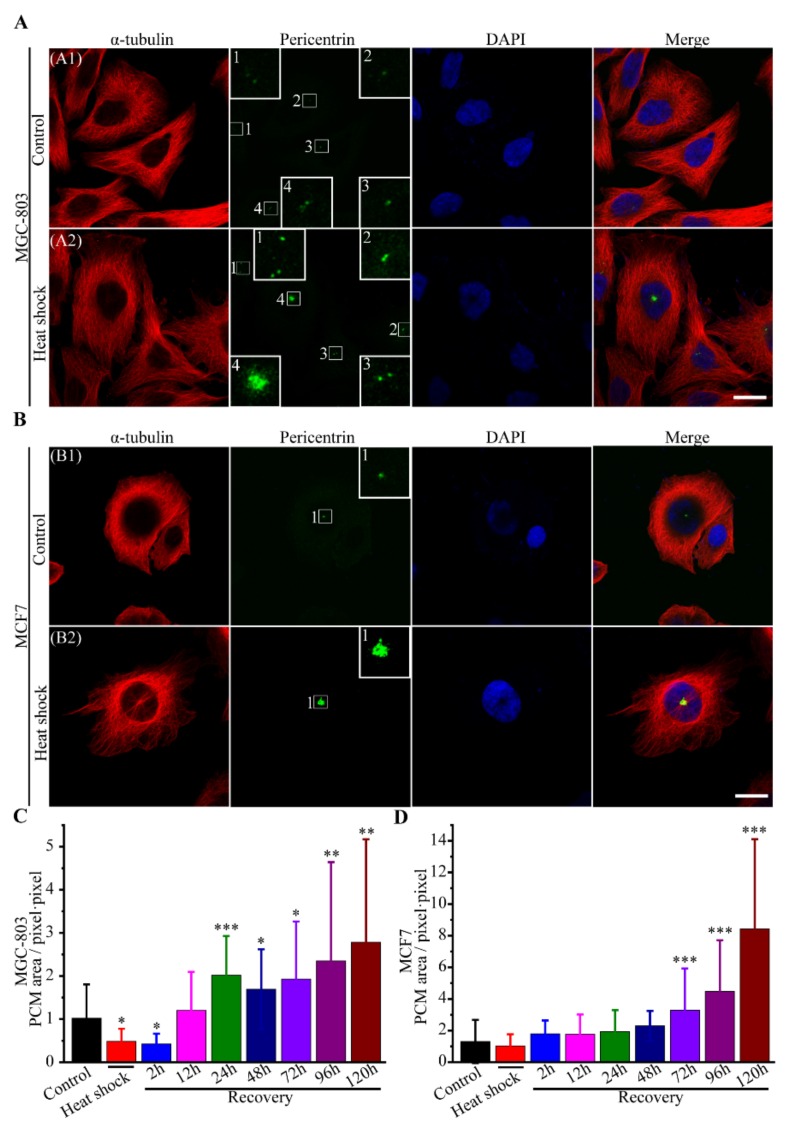
Pericentriolar material protein overexpression caused by heat stress. Immunofluorescence images stained with α-tubulin (red), pericentrin (green) antibodies and DNA (blue) of (**A**) MGC-803 cells and (**B**) MCF-7 cells, scale bar: 12 μm. (**C**,**D**) The relative areas of PCM in the immunofluorescence images of MGC-803 and MCF-7 cells, respectively (mean values ± SD are shown from three independent experiments with more than 50 cells). Centrosomes were often enlarged because of excessive PCM accumulation around centrioles after heat treatment. *p*-value < 0.05 was considered statistically significant, * *p* < 0.05, ** *p* < 0.01, and *** *p* < 0.001.

**Figure 6:**
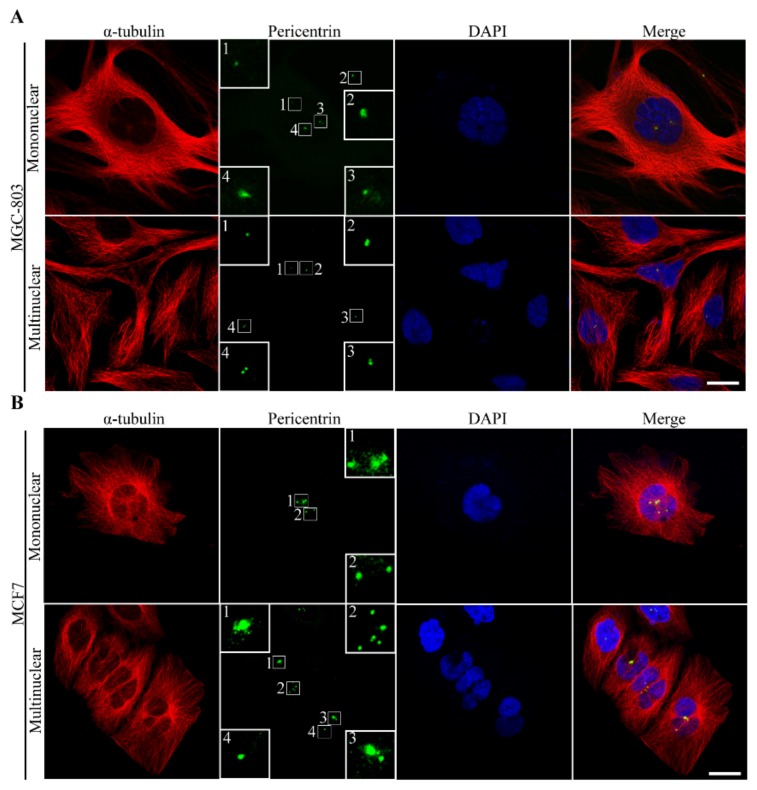
Multiple centrosomes in both mononuclear and multinuclear human cancer cells caused by heat stress. Immunofluorescence images stained with α-tubulin (red), pericentrin (green) antibodies and DNA (blue) of (**A**) MGC-803 cells and (**B**) MCF-7 cells, scale bar: 12 μm. Multiple foci of PCM were found in both mononuclear and multinuclear cells, implying supernumerary centrosomes induced by heat stress.

Heat stress can induce both structural and numerical aberrations of centrosomes in human cancer cells [15,17,20]. In our experiments, we first found that heat stress alters centrosome organization. Centrosomes appeared enlarged in size, due to the excessive PCM (pericentriolar material) accumulation around centrioles (Figure 5A,B). Furthermore, we tested the centrosome size by the area of PCM in the immunofluorescence images at different recovery durations after heat shock (Figure 5C,D). Our results revealed that the PCM protein in cancer cells was immediately degraded by heat stress, and became gradually overexpressed during recovery. In addition, supernumerary centrosomes (extra foci of PCM) were observed in both mononuclear and multinuclear cells (Figure 6A,B). It remains unclear whether the extra centrosomes derived from the direct centrosome self-replication by heat shock or the polyploid cells by previous mitotic slippage, cytokinesis failure and cell fusion. However, centrosome assembly is vital for mitotic spindle formation, that is, defects in centrosomes can lead to multiple spindle formation and facilitate multipolar divisions of cancer cells [46]. Using immunocytochemistry method, we validated the multipolar spindle formation in MGC-803 cells directly induced by heat shock, as shown in Figure S10.

#### 3.2.5. Incomes of Multiple Multipolar Divisions Induced by Heat Stress

**Figure 7:**
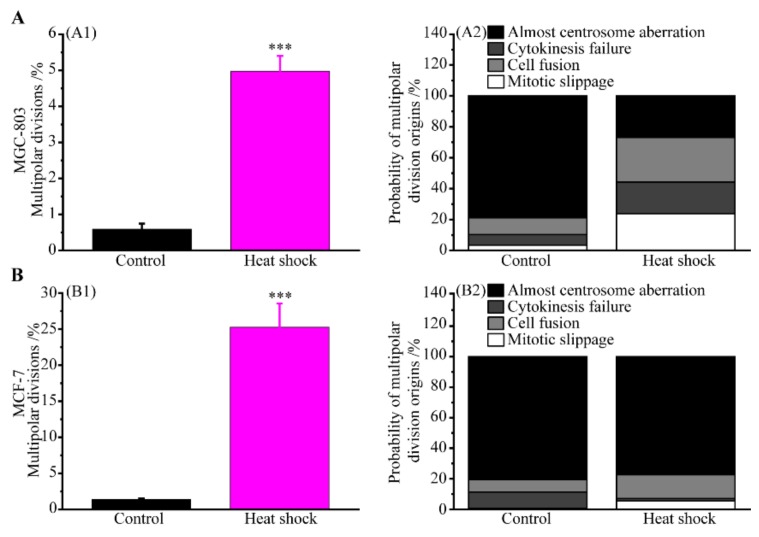
Multipolar divisions caused by heat stress and their probabilities originated from mitotic slippage, cytokinesis failure, cell fusion, and centrosome aberrations. (**A**) Multipolar divisions of MGC-803 cells; (**A1**) the multipolar divisions frequency of MGC-803 cells (mean values ± SD are shown from three independent experiments); (**A2**) the multipolar divisions probability originated from mitotic slippage, cytokinesis failure, cell fusion, and centrosome aberrations, respectively. (**B**) Multipolar divisions of MCF-7 cells; (**B1**) the multipolar divisions frequency of MCF-7 cells (mean values ± SD are shown from three independent experiments); (**B2**) the multipolar divisions probability originated from mitotic slippage, cytokinesis failure, cell fusion, and centrosome aberrations, respectively. *p*-value < 0.05 was considered statistically significant, *** *p* < 0.001.

Successful multipolar divisions of MGC-803 and MCF-7 cells were detected and counted after heat shock. Our results showed that 0.59% of MGC-803 cells and 1.37% of MCF-7 cells spontaneously underwent multipolar divisions, while the frequency was elevated to 4.98% and 25.26% after heat shock, respectively (Figure 7(A1,B1)). Moreover, we immediately traced the cells after heat stress through long-term live cell imaging and the origins of multiple multipolar divisions were experimentally determined (Figure 7(A2,B2)). In the control group, 3.4%, 6.9%, and 10.9% of multipolar divisions of MGC-803 cells originated from mitotic slippage, cytokinesis failure and cell fusion, respectively. By contrast, in the heated group, the contributions of previous mitotic slippage, cytokinesis failure and cell fusion to multipolar divisions of MGC-803 cells were elevated to 23.9%, 20.3%, and 28.9%, respectively. Additionally, 78.9% and 26.9% of multipolar divisions in MGC-803 cells in the control group and the heated group were caused by centrosome aberrations, respectively. In MCF-7 cells, 0.6%, 10.6%, and 8.1% of multipolar divisions were caused by previous mitotic slippage, cytokinesis failure and cell fusion, while the corresponding frequency was 5.5%, 1.5%, and 15.6% after heat shock, respectively. About 80.7% and 77.4% of multipolar divisions in MCF-7 cells in the control group and the heated group were due to centrosome aberrations, respectively. These results indicate that multipolar cell divisions induced by heat stress of human cancer cells are due to both the direct effect on cell centrosome aberrations and the indirect effect of the preceding polyploid cells generated by mitotic slippage, cytokinesis failure, or cell fusion. In addition, our experiments indicate that cell fusion is obviously the main trigger of the polyploidy formation of cancer cells induced by heat treatment, either for MGC-803 or MCF-7 cells.

## 4. Discussion

Spontaneous multipolar divisions are a rare event in human cancer cells under room temperature. By using long-term live-cell imaging, Ganem et al. [46] studied ten different cancer cell lines and found spontaneous multipolar divisions in the range from less 0.5% to about 9.5%, where the percentage of MCF-7 cells that spontaneously underwent multipolar divisions was less than 0.5%. Stewénius et al. [47] experimentally demonstrated multipolar mitoses in five colon cancer cell lines with a frequency of 1–4%. In the present study, multipolar divisions were detected in MGC-803 cells with a frequency of ~0.59% and MCF-7 cells with a frequency of ~1.37%, as shown in Figure 7(A1,B1). In addition, it has been found that both chemical substances and mechanical methods, such as paclitaxel, colchicine, carboplatin, cytochalasin, and even mechanically confined microenvironments, may induce or regulate multipolar divisions of cells with drastically higher frequencies. Bian et al. [8] treated HeLa cells with 4 nmol/L paclitaxel for 1 h and found, by staining with α-tubulin antibody, 80% of mitotic cells generated multipolar spindles. Tse et al. [12] applied confined mechanical environments to obtain the frequency of cell multipolar divisions of 50%, an astounding 50-fold increase from unconfined environments with less than 1%.

Heat stress is another kind of mechanical factor that can induce multipolar cell divisions [14,15,16,17,18,19,20]. For example, by immunofluorescence assay, Nakahata [17] et al. demonstrated the percentage of metaphases with multiple spindles of MCF-7 cells with about 3% in control and 71.0% in heat-shocked group. As shown in Figure 7(A1,B1), our results showed that the fractions of MGC-803 and MCF-7 cells that exhibited multipolar divisions after heat treatment of 48 °C for 10 min was elevated up to 4.98% and 25.26%, respectively. Centrosome aberrations of cells directly caused by heat stress has long been thought of as the mechanism underlying cell multipolar divisions induced by heat treatment. In this paper, we report several different biological routes which can render multipolar cell divisions due to heat shock. More specifically, heated human cancer cells may previously experience either mitotic slippage, cytokinesis failure, or cell fusion, resulting in polyploidy and, then, undergo multipolar divisions. Furthermore, after the heat shock, most dividing cells suffer mitotic slippage, while the cells in interphase tend to undergo cytokinesis failure and cell fusion. Meanwhile, cell centrosome aberrations of heated cancer cells were also evidenced with immunofluorescence observations, and the following formation of multipolar spindles were experimentally validated with MGC-803 cells and the outcome for multipolar divisions of heated cancer cells was also counted.

Considering mitotic slippages of heated cells, Westra et al. [29] revealed that the mitotic CHO cells exposed to heat shock can directly enter the next cell cycle without completing division and result in tetraploidy. Coss et al. [31] demonstrated that this abnormal mitosis was due to the disassembled spindle and destroyed contractile ring by heat stress. The contractile ring is a transient structure composed of actin, myosin II, and associated proteins [48,49,50]. Normally, cytokinesis has been considered to be accomplished by the contractile ring which generates contractile stresses to cleave the cell. However, using fluorescent microscopy, Glass et al. [51] revealed an immediate rapid loss of stress fibers (F-actin) in CHO cells after immersion in a 45 °C water bath. In addition, the effects of heat stress on cytoskeleton has been well reviewed by Coss et al. [52], showing that cell microtubule and microfilament systems are reorganized and the number of stress fibers is reduced, or even eliminated, after heat shock. In other words, heat shock may cause depolymerization of microtubules and microfilaments, leading to a disassembled spindle and destroyed contractile ring, and eventually resulting in mitotic slippages of cells.

Further, we detected the cells that have undergone the first divisions and cytokinesis failure after heat shock. A visible bridge can be observed between the daughter cells produced by the first cell divisions after heat stress (Figure S11). Hurwitz et al. [53] and Sasaki et al. [54] experimentally revealed that HeLa cells undergoing cell divisions after X-ray irradiation treatment were usually accompanied by the intercellular bridge formation to retract the separated cells into a binucleate cell. They confirmed that these bridges comprise chromatin and DNA fibers, suggesting that incomplete cell division occurs mainly as a consequence of chromosome or chromatin bridges. Furthermore, using long-term imaging experiments of HeLa cells, Shi et al. [55] found that lagging chromosomes are evident in the cytoplasmic bridge followed by furrow regression. Their results showed that chromosome nondisjunction directly generated tetraploid cells by cytokinesis failure that may subsequently become aneuploid through further divisions. Moreover, cytokinesis failure was followed by multipolar divisions in over half of the instances [53], which is consistent with our results in the present paper.

Fusion is normally a relatively rare but spontaneous event in both in vitro and in vivo cells [56,57,58]. Cell fusion has also been speculated to contribute to tumor initiation, malignancy, heterogeneity, drug resistance, and cancer stem cell origins [56,57,58,59,60,61]. For example, fusion efficiency can be proportional to the malignant level of tumor cells [56]. Our results revealed that heat stress can elevate cell fusion efficiency, and cell fusion is obviously the main contribution of the polyploidy formation of heated cancer cells, while the mechanism underlying heat-induced cell fusion remains unknown.

Multipolar spindle formation in the mitoses of cancer cells due to heat stress-induced centrosome aberrations have attracted considerable attentions [16,17,18]. In the present work, we have detected both the extra foci of PCM and the enlarged size of PCM after heat shock. Specifically, we find that the PCM protein is degraded soon after the heat stress and gradually overexpressed during the recovery. In particular, the PCM provides a scaffold for γ-tubulin ring complexes that play a key role in microtubule nucleation. By using γ-tubulin antibodies, Baek et al. [20] found that 85.7% of HeLa cells and 47.7% of NIH3T3 cells had no γ-tubulin spots after heat shock. During the recovery period, they found that γ-tubulin spots began to reappear and the percentage of cells with extra γ-tubulin spots increased. Moreover, because of PCM fragmentation, not a particle stained positive with anti-PCM antibodies represents a complete centrosome [2]. Although the effect of heat stress on centrosomes shows a similar tendency, heat stress can disassociate centrosomes, including both pericentrin and γ-tubulin, and then cause centrosome amplification.

As described above, mitotic slippage, cytokinesis failure, and cell fusion of cells can lead to cell polyploidies, which have supernumerary chromosomes and centrosomes compared with diploid cells. In addition, spindle multipolarity can be prevented by centrosomal clustering [62,63,64]. Therefore, these polyploid cells with supernumerary centrosomes can further undergo both bipolar and multipolar divisions. It has been found that the fate of polyploid cells differs from various cell lines. For example, approximate 52% of the tetraploid RPE-1 cells ultimately divide into two daughter cells, whereas 48% divide into three or more cells [7]. Unlike RPE-1 cells, 97% of binuclear HeLa cells form stable tri- or tetrapolar spindles that produce three or four daughter cells [7]. In the present study, we find that the fate of polyploid cells induced by heat shock depends strongly on their origins. First, polyploid cells (MGC-803 and MCF-7) produced by mitotic slippage after heat shock have less survival capacity compared with that those produced by cytokinesis failure and cell fusion. Second, in both the control and heated groups, the surviving MGC-803 and MCF-7 polyploid cells generated by mitotic slippage experience bipolar division at a higher rate than multipolar division, whereas polyploid cells generated by both cytokinesis failure and cell fusion are more likely to undergo multipolar divisions than bipolar divisions. Meanwhile, Vidair et al. [15] showed that the yielding multinucleated CHO cells induced by heat stress are apt to die. By contrast, our results show that the polyploid MGC-803 and MCF-7 cells can successfully survive and undergo next bipolar or multipolar divisions.

Finally, it is emphasized that there exist many factors that affect the multipolar divisions and subsequent fates of cells, which deserve further experimental and theoretical efforts. In addition, yet unclear are the molecular mechanisms that trigger the different biological routes for the polyploidy formation of heated cancer cells (mitotic slippage, cytokinesis failure, and cell fusion) and the subsequent multipolar divisions and fates of the induced polyploid cells.

## 5. Conclusions

In summary, we have provided a thorough and comprehensive compendium of multiple multipolar divisions of human cancer cells under heat stress. Our experiments demonstrate that heat stress can directly induce centrosome aberrations, thereby leading to multipolar divisions of cancer cells. In addition, it is found that heated human cancer cells can suffer polyploidizatoin by the abnormal biological responses, such as mitotic slippage, cytokinesis failure, or cell fusion, then undergo multipolar divisions. High frequency multiple multipolar divisions of cancer cells induced by heat stress usually generate aneuploid daughter cells, which might lead to tumor heterogeneity.

## Supplementary Materials

The supplementary materials are available online at https://www.mdpi.com/2073-4409/8/8/888/s1, Figure S1. Cancer cell shape change induced by heat stress at 48 °C for 10 min. (A) and (B), Changes in the spread areas of MGC-803 and MCF-7 cells, respectively. (C) and (D), Changes in the aspect ratios of MGC-803 and MCF-7 cells, respectively. Figure S2. Flow cytometry histograms of MGC-803 and MCF-7 cells at different recovery time points after heat treatment at 48 °C for 10 min. The flow cytometry distributions stained with propidium iodide (PI) of (A) MGC-803 cells and (B) MCF-7 cells. Figure S3. Cancer cells were in either interphase or mitosis when heat stress was applied. Images of (A) MGC-803 cells and (B) MCF-7 cells, where red arrowheads and yellow arrows indicate cancer cells in interphase and mitosis, respectively, scale bar: 100 μm. Figure S4. Mitotic slippage of cancer cells in interphase caused by heat stress and their cell fates. (A) Time-lapse images of MGC-803 cells, scale bar: 50 μm; (A1) bipolar division, (A2) multipolar division and (A3) cell apoptosis of MGC-803 cells after mitotic slippage induced by heat stress. (B) Time-lapse images of MCF-7 cells, scale bar: 40 μm; (B1) bipolar division, (B2) multipolar division and (B3) cell apoptosis of MCF-7 cells after mitotic slippage induced by heat stress. BP (bipolar), MP (multipolar), AP (apoptosis). Figure S5. The progeny fates of cancer cells only from bipolar divisions after mitotic slippage induced by heat stress. (A) Time-lapse images of MGC-803 cells, scale bar: 50 μm. (B) Time-lapse images of MCF-7 cells, scale bar: 40 μm. (C) Quantitative results of the progeny fates of MGC-803 cells from bipolar divisions after mitotic slippage induced by heat stress; we tracked 36 progenies from these MGC-803 cells and we found that 14, 9 and 13 of these daughter cells underwent bipolar divisions, multipolar divisions and cell apoptosis, respectively. Those corresponding progeny of MCF-7 cells was rarely found in our experiments and thus the quantitative results of their fates were not given here. BP (bipolar), MP (multipolar), AP (apoptosis). Figure S6. Cytokinesis failure of MGC-803 cells caused by heat stress and their cell fates. Time-lapse images of these cells where cytokinesis failures were induced by heat stress and then followed by (A) bipolar division, (B) multipolar division, (C) cell apoptosis, scale bar: 50 μm. Those corresponding MCF-7 cells was rarely observed in our experiments. BP (bipolar), MP (multipolar), AP (apoptosis). Figure S7. Cancer cell pairs and their viabilities after heat shock. Images of (A) MGC-803 and (B) MCF-7 cells, where yellow arrows indicate cancer cell pairs, scale bar: 100 μm. (C) Cell viability; here we detected 772 and 702 cell pairs of MGC-803 and MCF-7 cells after heat treatment, and we found that 605 and 273 cell pairs of these cancer cells were kept alive, respectively. Figure S8. Cell division processes of cancer cell pairs without cell fusion in the control groups. Time-lapse images of (A) MGC-803 cell pairs (scale bar: 50 μm) and (B) MCF-7 cell pairs (scale bar: 40 μm). Figure S9. Duration time distribution of cell fusion of (A) MGC-803 and (B) MCF-7 cells. The average duration time of cell fusion was 123.5 min for MGC-803 cell pairs and 46.9 min for MCF-7 cell pairs. Figure S10. Immunofluorescence images of MGC-803 cells, stained with α-tubulin (red), pericentrin (green) and DNA (blue). Image galleries were acquired at about 0.8 μm intervals on the *Z*-axis of (A) a bipolar mitosis with two foci of PCM in the control cells and (B) a tripolar mitosis with four foci of PCM in the heated cells, scale bar: 5 μm. Numbers “1-15” in the immunofluorescence images indicate the different stacks. Figure S11. Viable bridges between two daughter cancer cells with cytokinesis failure. (A) Time-lapse images of MGC-803 cells; (A1) visible bridge was observed between the daughter cells underdoing cytokinesis failure in MGC-803 cells; (A2) and (A3) are the enlarged views of part of the first and second images in (A1), respectively. (B) Time-lapse images of MCF-7 cells; (B1) visible bridge was observed between the daughter cells underdoing cytokinesis failure in MCF-7 cells; (B2) and (B3) are the enlarged views of part of the first and second images in (B1), respectively. Table S1. The STR profile of MGC-803 cell line.
