# Supplementary material for: Heat Stress-Induced Multiple Multipolar Divisions of Human Cancer Cells
Source: Cells. 2019 Aug 13;8(8):888. doi: 10.3390/cells8080888 (PMC6721694; doi:10.3390/cells8080888)
Supplement: Supplementary file 1 [file cells-08-00888-s001.pdf]

Supplementary Materials

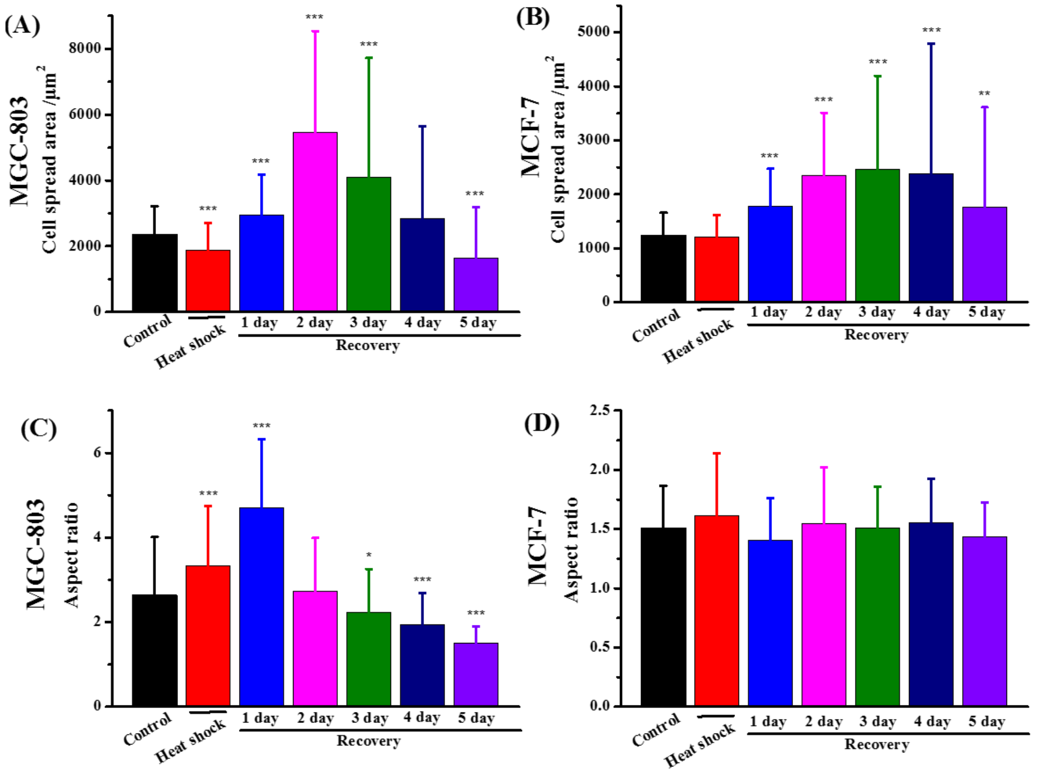

Figure S1. Cancer cell shape change induced by heat stress at 48 °C for 10 min. (**A**) and (**B**), Changes in the spread areas of MGC-803 and MCF-7 cells, respectively. (**C**) and (**D**), Changes in the aspect ratios of MGC-803 and MCF-7 cells, respectively.

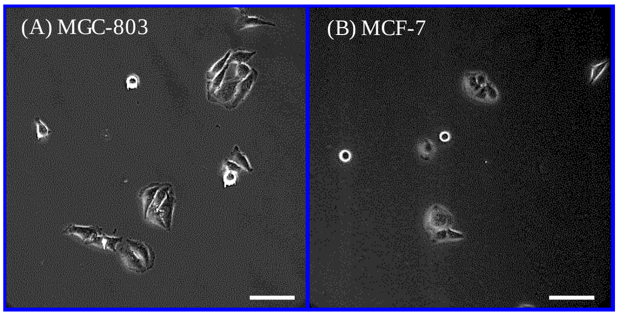

Figure S2. Flow cytometry histograms of MGC-803 and MCF-7 cells at different recovery time points after heat treatment at 48 °C for 10 min. The flow cytometry distributions stained with propidium iodide (PI) of (**A**) MGC-803 cells and (**B**) MCF-7 cells.

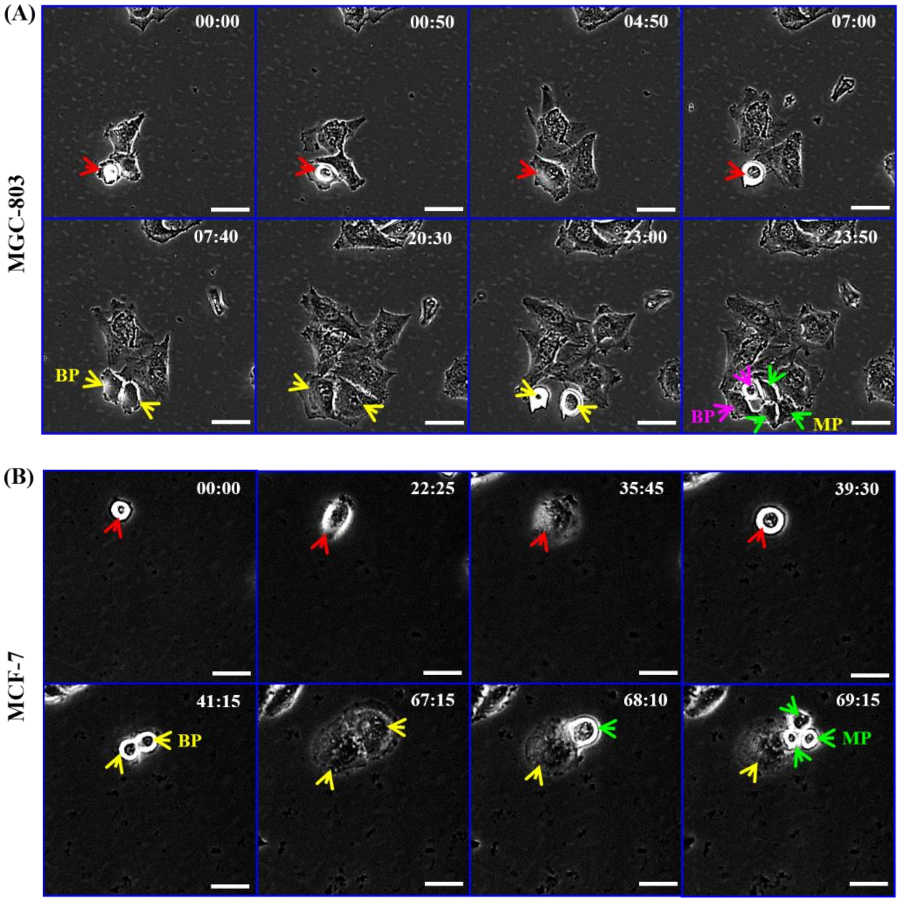

Figure S3. Cancer cells were in either interphase or mitosis when heat stress was applied. Images of (**A**) MGC-803 cells and (**B**) MCF-7 cells, where red arrowheads and yellow arrows indicate cancer cells in interphase and mitosis, respectively, scale bar: 100 μm.

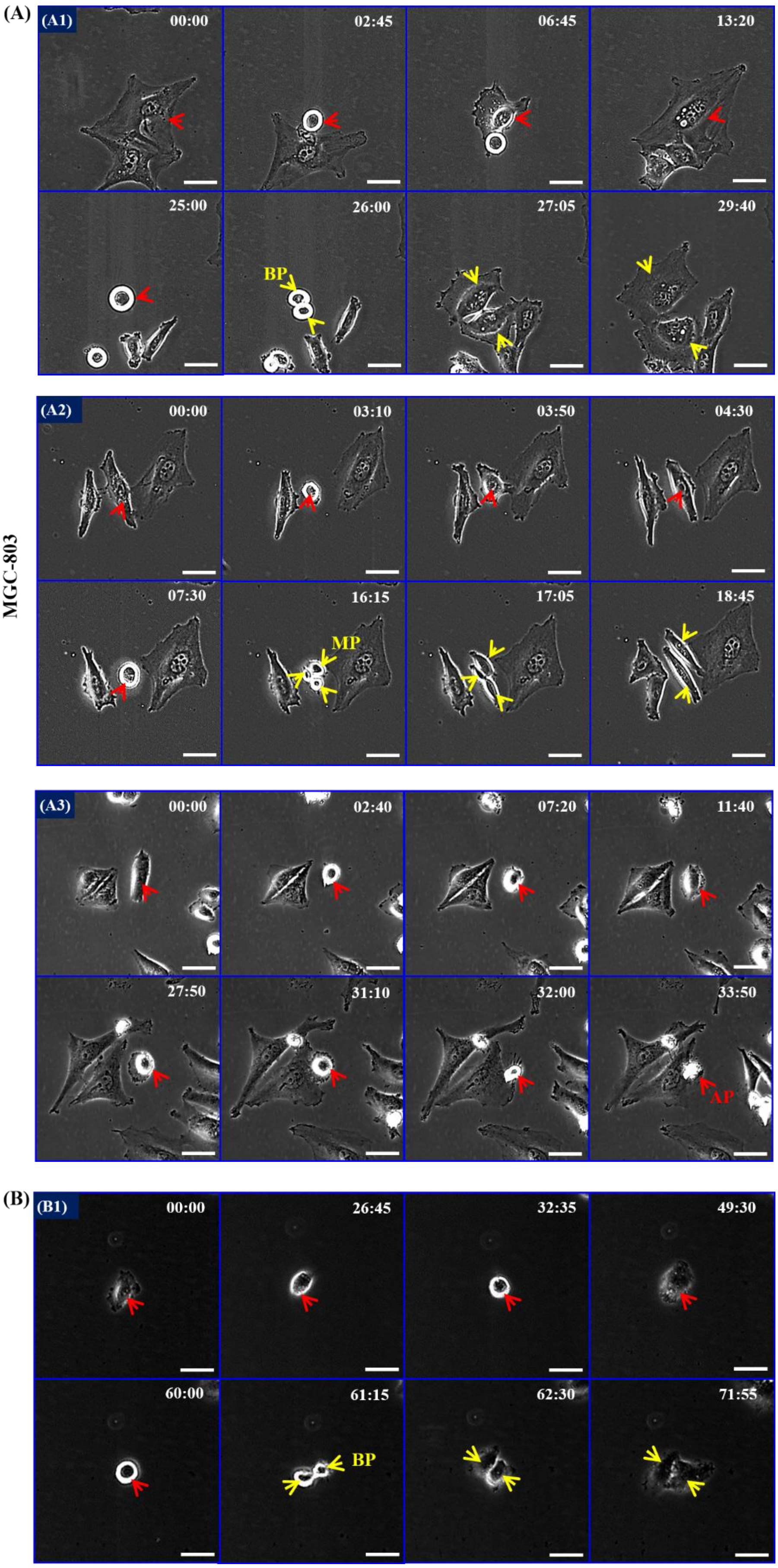
(A)
(A1)
00:00
02:45
06:45
13:20
25:00
26:00
27:05
29:40
BP
MGC-803
(A2)
00:00
03:10
03:50
04:30
07:30
16:15
17:05
18:45
MP
(A3)
00:00
02:40
07:20
11:40
27:50
31:10
32:00
33:50
AP
(B)
(B1)
00:00
26:45
32:35
49:30
60:00
61:15
62:30
71:55
BP

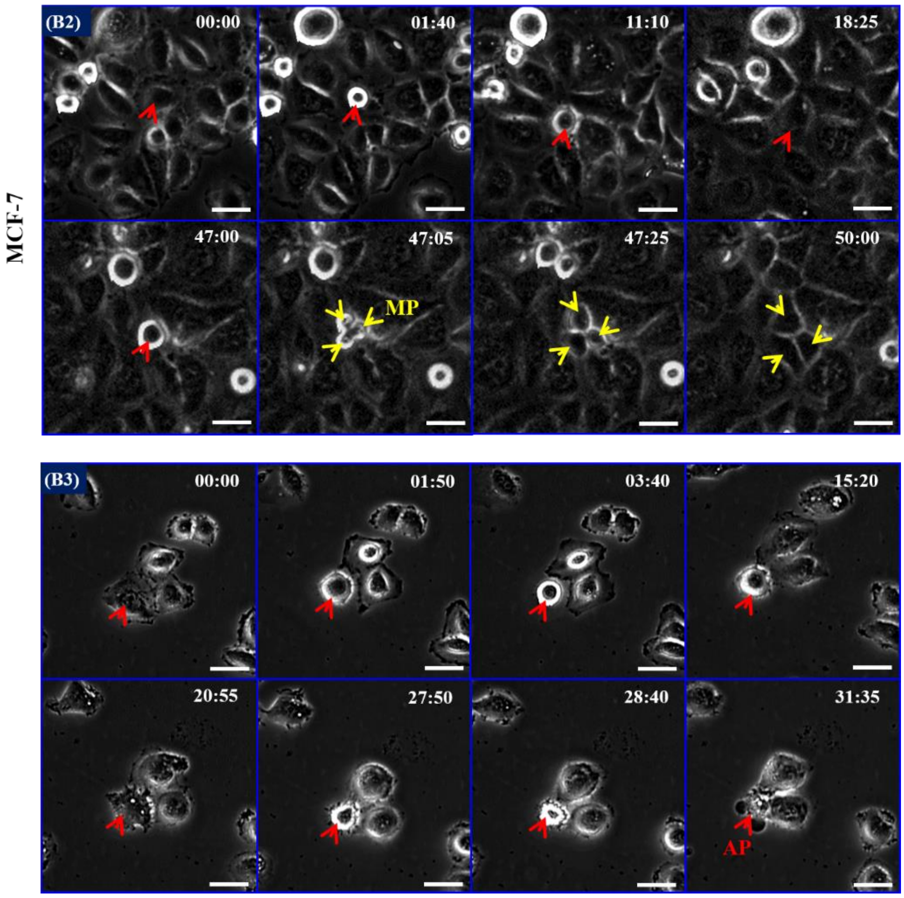

Figure S4. Mitotic slippage of cancer cells in interphase caused by heat stress and their cell fates. (**A**) Time-lapse images of MGC-803 cells, scale bar: 50 µm; (**A1**) bipolar division, (**A2**) multipolar division and (**A3**) cell apoptosis of MGC-803 cells after mitotic slippage induced by heat stress. (**B**) Time-lapse images of MCF-7 cells, scale bar: 40 µm; (**B1**) bipolar division, (**B2**) multipolar division and (B3) cell apoptosis of MCF-7 cells after mitotic slippage induced by heat stress. BP (bipolar), MP (multipolar), AP (apoptosis).

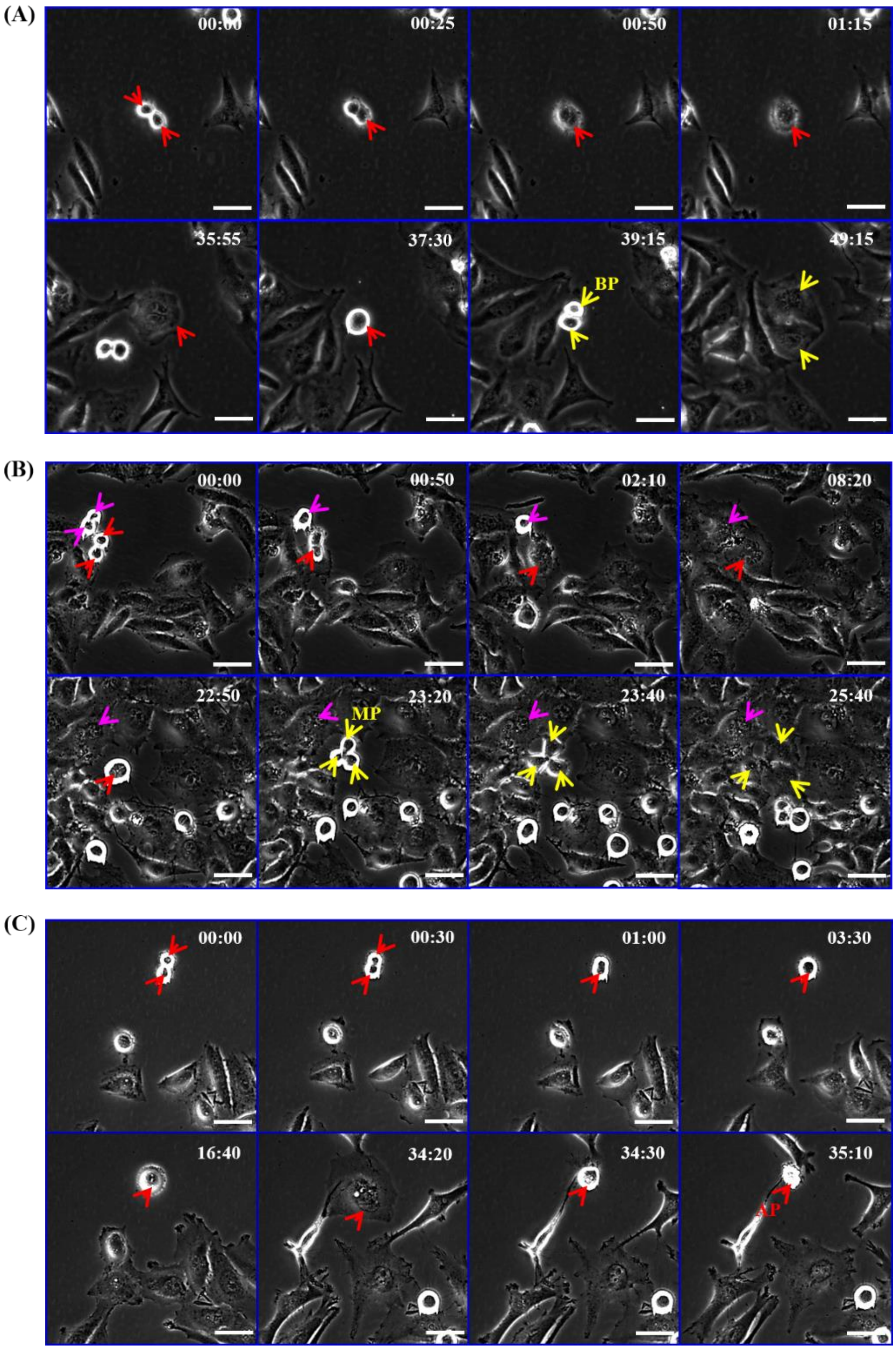

Figure S5. The progeny fates of cancer cells only from bipolar divisions after mitotic slippage induced by heat stress. (**A**) Time-lapse images of MGC-803 cells, scale bar: 50 µm. (**B**) Time-lapse images of MCF-7 cells, scale bar: 40 µm. (**C**) Quantitative results of the progeny fates of MGC-803 cells from bipolar divisions after mitotic slippage induced by heat stress; we tracked 36 progenies from these MGC-803 cells and we found that 14, 9 and 13 of these daughter cells underwent bipolar divisions, multipolar divisions and cell apoptosis, respectively. Those corresponding progeny of

MCF-7 cells was rarely found in our experiments and thus the quantitative results of their fates were not given here. BP (bipolar), MP (multipolar), AP (apoptosis).

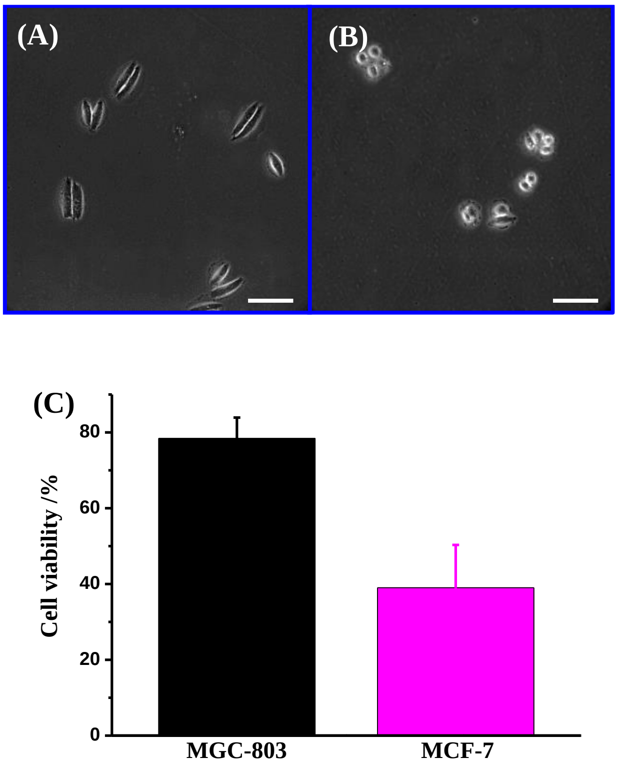

Figure S6. Cytokinesis failure of MGC-803 cells caused by heat stress and their cell fates. Time-lapse images of these cells where cytokinesis failures were induced by heat stress and then followed by (**A**) bipolar division, (**B**) multipolar division, (**C**) cell apoptosis, scale bar: 50 μm. Those corresponding MCF-7 cells was rarely observed in our experiments. BP (bipolar), MP (multipolar), AP (apoptosis).

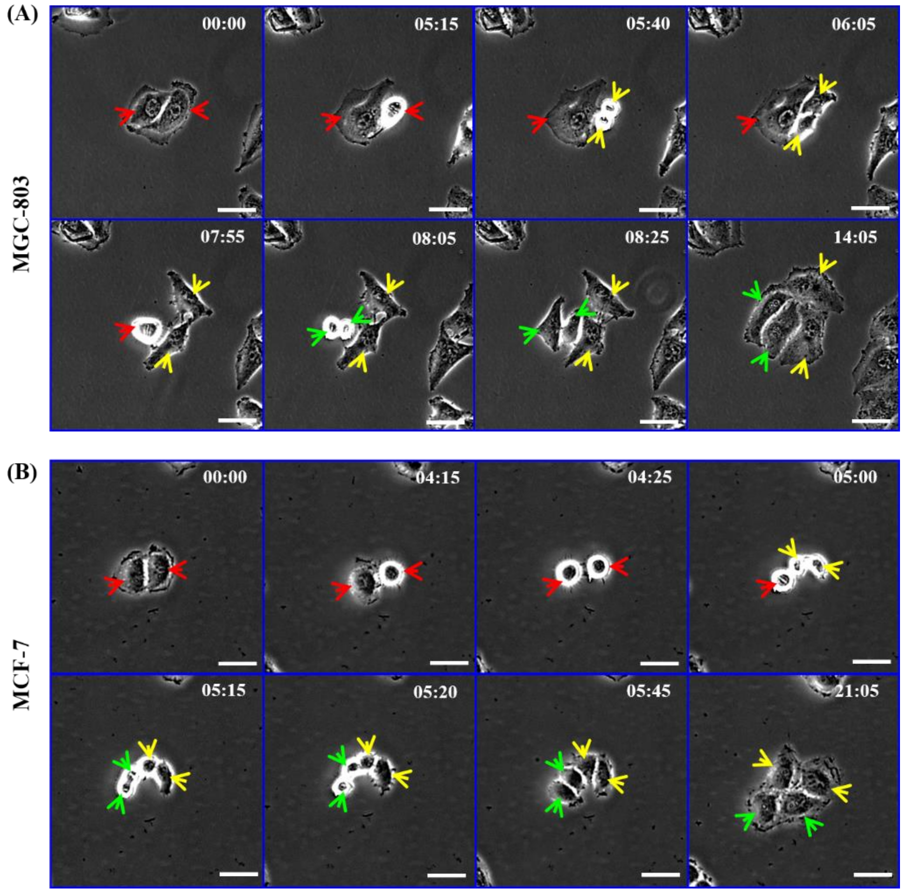

Figure S7. Cancer cell pairs and their viabilities after heat shock. Images of (A) MGC-803 and (**B**) MCF-7 cells, where yellow arrows indicate cancer cell pairs, scale bar: 100 μm. (**C**) Cell viability; here we detected 772 and 702 cell pairs of MGC-803 and MCF-7 cells after heat treatment, and we found that 605 and 273 cell pairs of these cancer cells were kept alive, respectively.

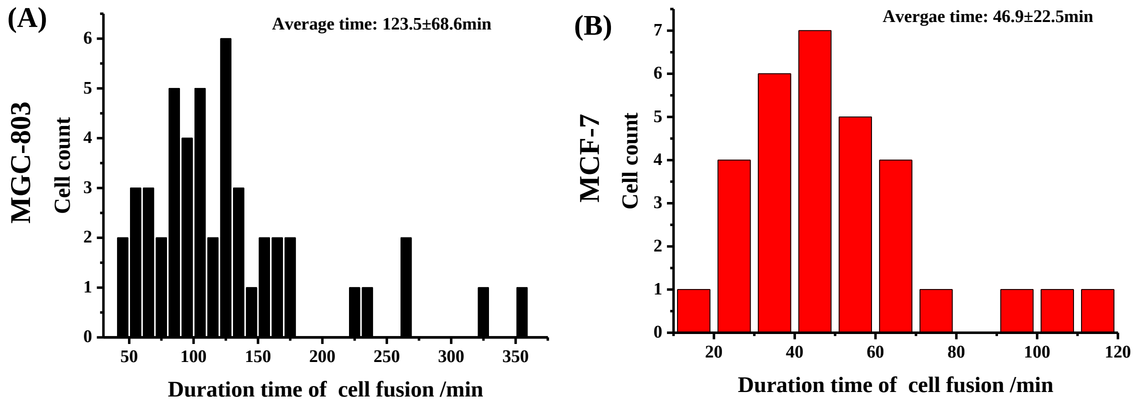

Figure S8. Cell division processes of cancer cell pairs without cell fusion in the control groups. Time-lapse images of (**A**) MGC-803 cell pairs (scale bar: 50 μm) and (**B**) MCF-

7 cell pairs (scale bar: 40 μm).

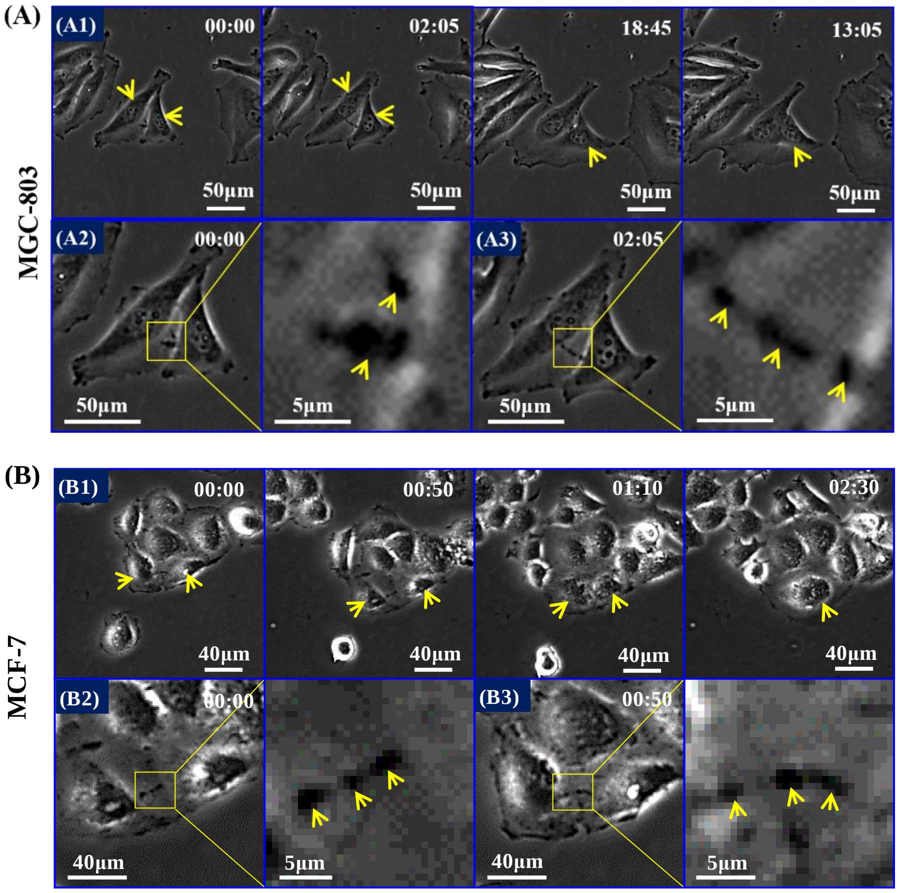

Figure S9. Duration time distribution of cell fusion of (**A**) MGC-803 and (**B**) MCF-7 cells. The average duration time of cell fusion was 123.5 min for MGC-803 cell pairs and 46.9 min for MCF-7 cell pairs.

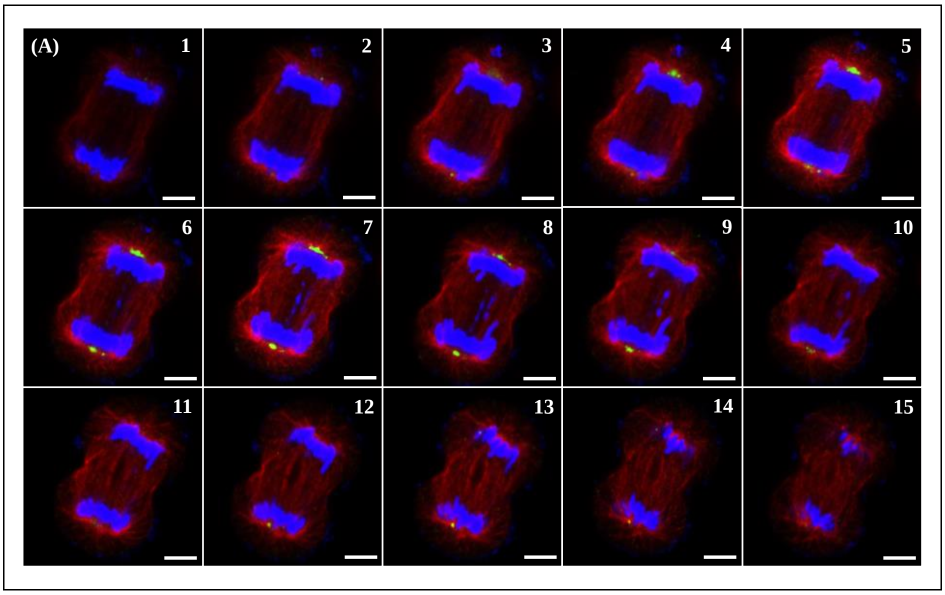

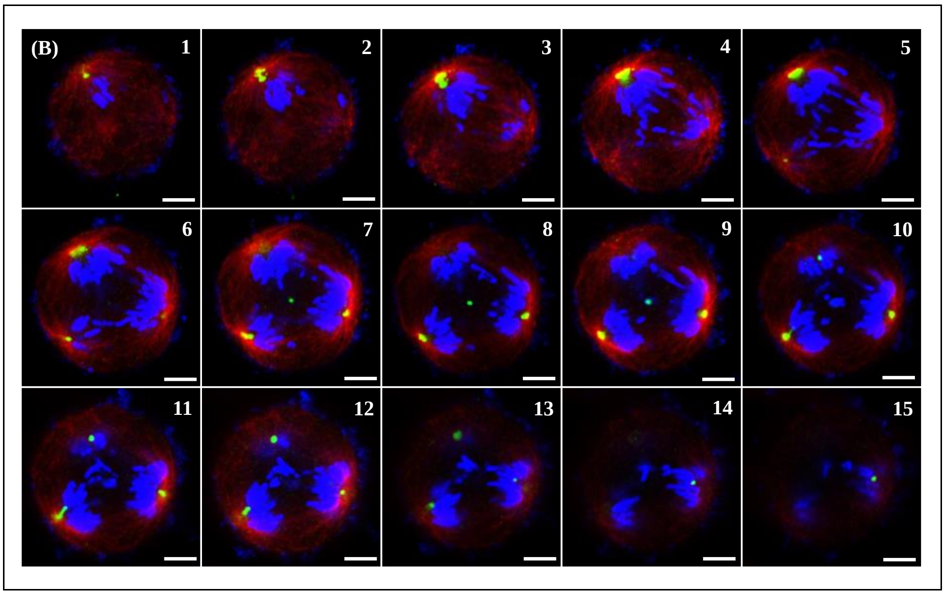

Figure S10. Immunofluorescence images of MGC-803 cells, stained with α-tubulin (red), pericentrin (green) and DNA (blue). Image galleries were acquired at about 0.8 μm intervals on the Z-axis of (**A**) a bipolar mitosis with two foci of PCM in the control cells and (**B**) a tripolar mitosis with four foci of PCM in the heated cells, scale bar: 5μm. Numbers "1–15" in the immunofluorescence images indicate the different stacks.

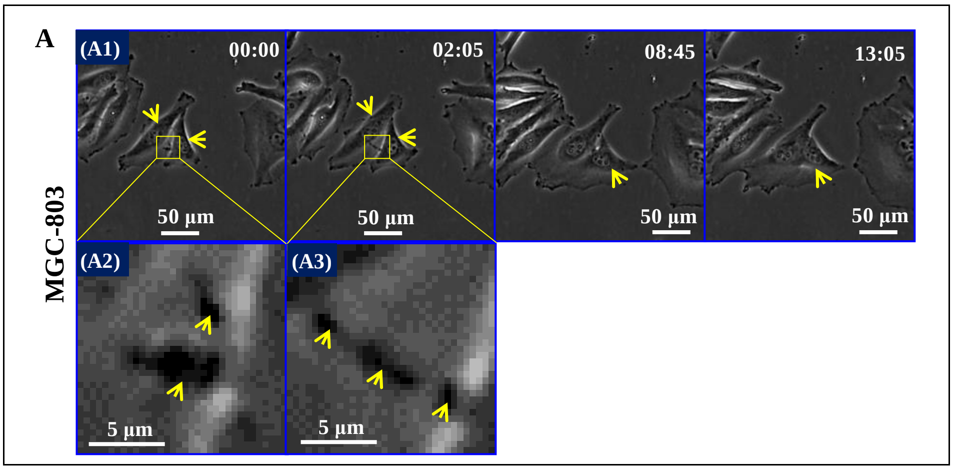

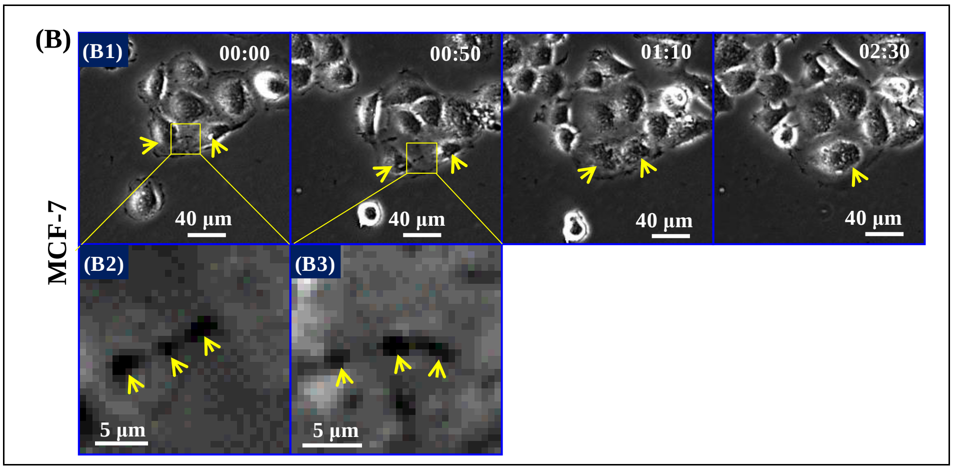

Figure S11. Viable bridges between two daughter cancer cells with cytokinesis failure. (**A**) Time-lapse images of MGC-803 cells; (**A1**) visible bridge was observed between the daughter cells underdoing cytokinesis failure in MGC-803 cells; (**A2**) and (**A3**) are the enlarged views of part of the first and second images in (**A1**), respectively. (**B**) Time-lapse images of MCF-7 cells; (**B1**) visible bridge was observed between the daughter cells underdoing cytokinesis failure in MCF-7 cells; (B2) and (B3) are the enlarged views of part of the first and second images in (**B1**), respectively.

Table S1. The STR profile of MGC-803 cell line.

	MGC-803 cells
STR	Amelogenin:X; CSF1PO:9, 10, 12; D13S317:7, 13.3; D16S539:9, 11; D18S51:13; D19S433:13; D21S11:27, 28; D2S1338:17, 24; D3S1358:15, 18; D5S818:10, 11, 12; D7S820:11, 12; D8S1179:12, 14; FGA:18, 21; TH01:7, 9; TPOX:12; vWA:16, 17, 18;
